# Multiplex detection of meningitis and encephalitis pathogens: A study from laboratory to clinic

**DOI:** 10.3389/fneur.2022.1054071

**Published:** 2022-12-16

**Authors:** Yanjun Si, Weijun He, Shuo Guo, Xiaohui Wang, Meng Tang, Binwu Ying, Minjin Wang

**Affiliations:** ^1^Department of Laboratory Medicine, West China Hospital of Sichuan University, Chengdu, Sichuan, China; ^2^Department of Clinical Laboratory, Chengdu Women's and Children's Central Hospital, School of Medicine, University of Electronic Science and Technology of China, Chengdu, Sichuan, China; ^3^Center of Infectious Diseases, West China Hospital of Sichuan University, Chengdu, Sichuan, China; ^4^Center for Infectious Diseases, Yaan People's Hospital, Yaan, Sichuan, China

**Keywords:** encephalitis, meningitis, multiplex PCR assay, X-pert MTB/RIF, CNS infection

## Abstract

**Introduction:**

Infectious meningitis and encephalitis (ME) are life-threatening conditions are caused by various pathogens. Conventional laboratory tests with low sensitivity and specificity cannot help with early diagnosis.

**Methods:**

A prospective study using the novel multiplex PCR detection for 18 pathogens of ME (MME-18) was conducted to investigate the clinical utilization and the epidemiology characteristics of ME in southwestern China. Patients with suspected intracranial infection were recruited between May and October 2019 at West China Hospital of Sichuan University. The MME-18 was used to detect cerebrospinal fluid, and conventional experiments including cryptococcal capsular antigen detection, GeneXpert, real-time PCR, and clinical feedback were used to verify the result of MME-18.

**Results:**

Among 581 tested patients, 139 eligible individuals were enrolled in the study. Among them, *Mycobacterium tuberculosis* was the most common pathogen in mono-infection. Viruses and *Cryptococcus neoformans* were also frequently detected. Of 139 infected patients, 12 cases were diagnosed by MME-18 only, 57 patients by conventional testing only, and 70 cases by both comparator tests and MME-18. There were 96.3% (79/82) diagnoses made by MME-18 had a favorable outcome, and two of twelve diagnoses, made solely by MME-18, had a likely unclear clinical significance.

**Discussion:**

The MME-18 showed satisfactory consistency with expert clinical consensus for patients presenting with ME. Combined with conventional testing and clinical suspicion, MME-18 may help clinicians with the early identification of pathogens.

## Introduction

Meningitis and encephalitis (ME), caused by pathogenic microorganisms, are the inflammation of infected meninges and brain parenchyma, respectively. They are severe infectious diseases with substantial morbidity, and devastating outcomes for individuals, families, and communities. Based on the 2019 Global Burden of Disease (GBD) study, except for tuberculous and cryptococcal meningitis cases, ME led to over 236,000 deaths worldwide, with over 2.5 million new cases of bacterial meningitis (1). It has been estimated that 2.6 per 100,000 adults developed bacterial meningitis in developed countries (2), and the incidence rate of viral and fungal meningitis was higher (3, 4). Up to 51% of cases of ME were from less-developed regions (5). Despite antimicrobial therapies and supportive care, the prognosis of ME is still poor. Complications such as focal neurological deficits, hearing loss, cognitive impairment, and epilepsy occur in nearly 50% of cases (2, 6, 7). Early recognition of the causative microorganisms allows early therapeutic intervention.

The causative pathogens of ME include viruses, bacteria, or other microorganisms, while the main causative pathogen widely differs from country and region. In Asia, Japanese encephalitis virus (JEV), *Streptococcus pneumoniae* (SP), and *Mycobactererium tuberculosis* (MTB) are frequently detected (8–10), but in American and Europe, patients are often infected by *Haemophilus influenzae* (HI) (11, 12). In Africa, cryptococcal meningitis is a critical public health problem (3). Effective antimicrobial treatment markedly varies for different pathogens (13). One study conducted a comprehensive surveillance of acute meningitis or encephalitis among 20,454 Chinese patients between 2009 and 2018. The study showed that herpes simplex virus (HSV) and JEV were the most two frequently determined viruses in adulthood ME (14). Tseng et al. (15) reported a positive rate of 7.5% for pathogens among 443 patients with suspected ME in 2021, in Taiwan. The study found that 60.6% of the detected pathogens are viruses, including varicella-zoster virus (VZV) in seven cases, herpes simplex virus-2 (HSV-2) in five cases, and human herpesvirus-6 (HHV-6) in three cases, human parechovirus in four cases, and herpes simplex virus-1 (HSV-1) in 1 case. However, due to the limited number of studies, we still know little about the common pathogens and incidence of ME patients in Southwestern China. Thus, investigating the epidemiologic features of pathogens among the Chinese population is crucial for clinicians in this area to adopt prompt and appropriate antimicrobial treatment.

The diagnosis of ME is always challenging for clinicians due to non-specific manifestations. Generally, the identification of pathogens heavily relies heavily on laboratory testing to detect pathogens in cerebrospinal fluid (CSF) (16, 17). Although cytology and biochemical analysis of CSF combined with conventional microbiological culture and serological methods may suggest the causative organisms, they are not sufficient as the only diagnostic criteria (18). Besides, microorganism cultures are the gold standard for diagnosing infectious diseases. However, the application of culture is limited by the late response and fastidious conditions in viral culture (19, 20) or paucibacillary culture of MTB (21). Recently, molecular techniques, including real-time PCR (5), 16S/23S rRNA gene amplification (22), and next-generation sequencing (23), have been introduced and widely used in clinics. However, due to limited detection throughput and time-consuming data analysis, their use has been unsatisfactory in clinical practice.

Multiplex PCR is a PCR amplification technique that can simultaneously and quickly detect various pathogens in a PCR reaction system. It has been reported that multiplex PCR methods, such as FilmArray^®^ Meningitis/Encephalitis Panel (FilmArray ME Panel, BioFire Diagnostics, bioMérieux, Marcy l'Etoile, France), are a sensitive and specific diagnostic tool for infectious meningitis and encephalitis (24–26). In this study, a novel Multiplex PCR detection for ME panel (MME-18) was designed to identify 18 common pathogens in CSF, including *Neisseria meningitidis* (NM), MTB, HI, *Mycoplasma pneumoniae* (MP), SP, *Acinetobacter baumannii* (AB), *Streptococcus agalactiae* (GBS), *Escherichia coli K1* (*E.coli* K1), *Listeria monocytogenes* (LM), *Cryptococcus neoformans* (CN), enterovirus (EV), mumps virus (MuV), HSV-1, HSV-2, VZV, Epstein-Barr virus (EBV), cytomegalovirus (CMV), and HHV-6. The procedure details have been reported previously (27), but its utilization for MME-18 has not been investigated. Therefore, this study investigated the epidemiological characteristics of ME pathogens in Southwestern China using the novel detection method. This method can help clinicians achieve early and rapid differentiation and recognition of pathogens in CSF.

## Materials and methods

### Study participants and clinical specimens

A prospective study was conducted at West China Hospital and enrolled patients from 13 cities in Southwestern China for 6 months (from May to October 2019). The likelihood of central nervous system infection for all cases and whether cases fit Brighton diagnostic criteria for meningitis, encephalitis, or meningoencephalitis were determined by physicians. Individuals meeting the following inclusion criteria were selected:

1. Patients were admitted to the Emergency, Neurology, and Infectious Diseases Department and presented with at least one of the following symptoms: (1) altered level of consciousness, (2) fever, (3) seizure, (4) focal neurological findings, (5) electroencephalographic or neuroimaging findings consistent with encephalitis or meningitis, and (6) refractory headaches;
2. CSF was collected before antimicrobial treatment or immunotherapy;
3. Thorough clinical information was obtained;
4. At least 3 mL of fresh and unpolluted (CSF) samples were collected by a lumbar puncture;
5. Nucleic acid extraction was performed within 2 h with an adequate residual volume of uncentrifuged (>1 mL) after the bacterial culture.

**Figure 1:**
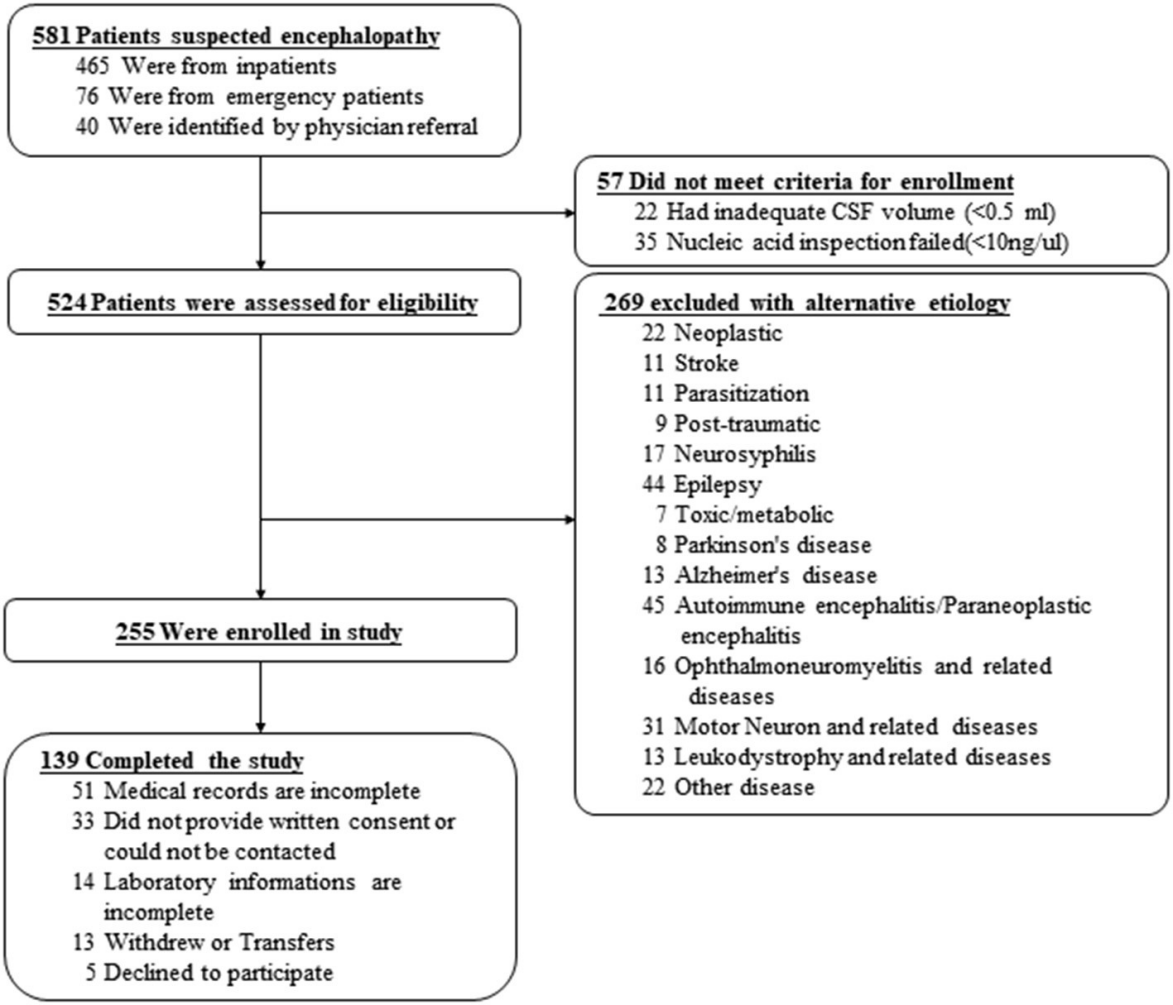
The flowchart of the enrollment.

All excess CSF samples were kept in a −80°C refrigerator, and repeat specimens from the same subject were excluded. Each sample used for this study was assigned a specific code number before Multiplex PCR detection. Besides, patients with another diagnosis and other complications were excluded (Figure 1). A total of 581 patients who were suspected to have ME were recruited from West China Hospital, Sichuan University. After excluding patients with definitive diagnoses—including neoplasm, stroke, Parasitic infection, brain trauma, neurosyphilis, epilepsy, toxic/metabolic encephalopathy, Parkinson's disease, Alzheimer's disease, autoimmune encephalitis, paraneoplastic encephalitis, ophthalmoneuromyelitis and related diseases, motor neuron and related diseases, and leukodystrophy and related diseases-−139 cases were finally included in the study, with the positive result of at least 1 pathogen among 18 tested pathogens by the combination of MME-18, conventional testing, and clinical evaluation. Patients with incomplete clinical documents were excluded from the study. The Institutional Review Board of the WestChina Hospital (201908032WCH) approved this study and informed consent was signed by all study participants.

### Nucleic acid extraction and MME-18 testing

Total nucleic acids (DNA/RNA) were extracted from approximately 1 mL CSF specimens using the nucleic acid extraction kit (Ningbo Health Gene Technology, Ningbo, China) on the automated extraction workstation Smart LabAssist-16/32 (Taiwan Advanced Nanotech Inc, Taiwan), according to the manufacturer's instructions. The nucleic acid was subjected to multiplex amplification for all specimens using Meningitis/Encephalitis Pathogen Multiplex Detection Kit (Ningbo Health Gene Technology, Ningbo, China) on ABI Verity 96 Thermal Cycler (Thermo Fisher Scientific, Carlsbad, CA, USA). The PCR product was subjected to capillary electrophoresis and fragment analysis using a 3500Dx Genetic Analyzer (Thermo Fisher Scientific, Carlsbad, CA, USA) according to the manufacturer's protocol (Supplementary Figure S1). The procedure details had been reported previously (27). The panel was designed to detect 18 pathogens within 4 h.

### Clinical information and adjunctive testing

#### Clinical information

Clinical data from enrolled patients about their sex, age, admitted department, initial symptoms, on-admission diagnosis, on-discharge diagnosis, adjunctive testing results, and medications were fully recorded and used for evaluating the accuracy of MME-18 coupled with the following adjunctive testing.

#### CSF bacterial and fungal culture testing

CSF samples from each patient included in the study were tested for bacterial culture. Tests were performed according to the laboratories' standard operating procedures of the Department of Laboratory Medicine, West China Hospital, Sichuan University. Liquid media (thioglycolate broth medium for increased bacteria culture) and solid media (Rabbit blood, Chocolate, Sabouraud's, Loewenstein-Jenson, and in some cases MacConkey medium) were incubated at 35 to 37°C in 5% CO_2_ for 2 to 5 days. Bacterial isolates were identified by standard procedures such as biochemical, phenotypic, or matrix-assisted laser desorption ionization-time of flight mass spectroscopy [MALDI-TOF MS] analysis.

Gram staining and Black-Ink staining were performed with cytocentrifuged CSF. At least two laboratory technologists reviewed the results of Gram staining and organisms isolated in culture or that did not yield any growth.

#### Sequencing and other comparator tests

Select the same nucleic acid sample as MME-18. Each positive viral result sample with sequencing comparator assays (Sangon Biotech, Shanghai, China) was conducted. Information for all of the primers has been listed in the previous report (27). The sequencing data were compared with the data provided by NCBI to confirm the pathogen.

The MME-18 results identified patients who are positive for MTB needs to use a CSF sample (>1 mL) obtained from the same time of MME-18 to be detected with Xpert MTB/RIF as comparator assays. The Xpert MTB/RIF (Cepheid, Sunnyvale, CA) was performed using a 100-fold dilution of the 1.0 McFarland standard suspension, and the results were interpreted according to the instruction.

The patients detected positive for CN were verified by cryptococcal capsular antigen detection. The cryptococcal capsular antigen detection kit was read by CrAg Lateral Flow Assay (IMMY, USA).

### Clinician feedback

After confirming the results, preliminary feedback was given to the attending physicians to help with the initial diagnosis and treatment. A summary report was prepared after obtaining all results. Our researchers collaborated with attending physicians, through interviews or telephone conversations on a weekly, to identify clinical diagnosis results and treatment progress. These clinical data were fully recorded and used for retrospective comparative analysis. All clinical data and clinician feedback were continuously collected until discharged or the treatment was completed. Telephone follow-up was performed 1 month after hospital discharge.

## Results

### Clinical characteristics of enrolled patients

A total of 139 cases were enrolled in the study based on the inclusion and exclusion criteria (Figure 1). The majority of CSF samples (94.6%) were from hospitalized patients. Patients admitted to the emergency department, and outpatients accounted for 4.1 and 1.3%, respectively. The average age of included patients was 44.9 years old, of which 57.6% (80/139) were men. As shown in Table 1, six patients were immunocompromised, including 4 with HIV-1 infection and 2 with organ transplantation. Patients with encephalitis with or without meningitis accounted for 60.4% (84/139), 38.1% (53/139) of all enrolled patients developed only meningitis, and 20.9% (29/139) experienced exacerbation. Among all patients, 12.2% (17/139) required intensive care, while only one patient died within 30 days after admission. CSF samples were collected from all patients within an average time of 2.2 days.

**Table 1:** Demographic and clinical characteristics of the 139 patients.

**Characteristic**	**Value**
**Age**	
Mean—yr	44.9
10–19 yr	9 (6.5)
20–39 yr	51 (36.7)
40–59 yr	45 (32.4)
>60 yr	34 (24.4)
Male sex — no. (%)	80 (57.6)
Meningitis alone	53 (38.1)
Encephalitis with or without meningitis	84 (60.4)
Myelitis with or without meningitis	2 (1.5)
Exacerbation of chronic condition—no. (%)	29 (20.9)
Immunocompromised—no. (%)	6 (4.0)
HIV-1	4 (2.7)
Solid-organ transplant	2 (1.4)
Immunosuppression for non-neoplastic condition	6 (4.3)
ICU admission—no. (%)	17 (12.2)
Death within 30 days—no. (%)	1 (0.7)
Median no. of days after hospital admission that CSF was collected for MME-18 assay (range)—days	2.2 (1–8)

### Identification of potentially causative pathogens

**Figure 2:**
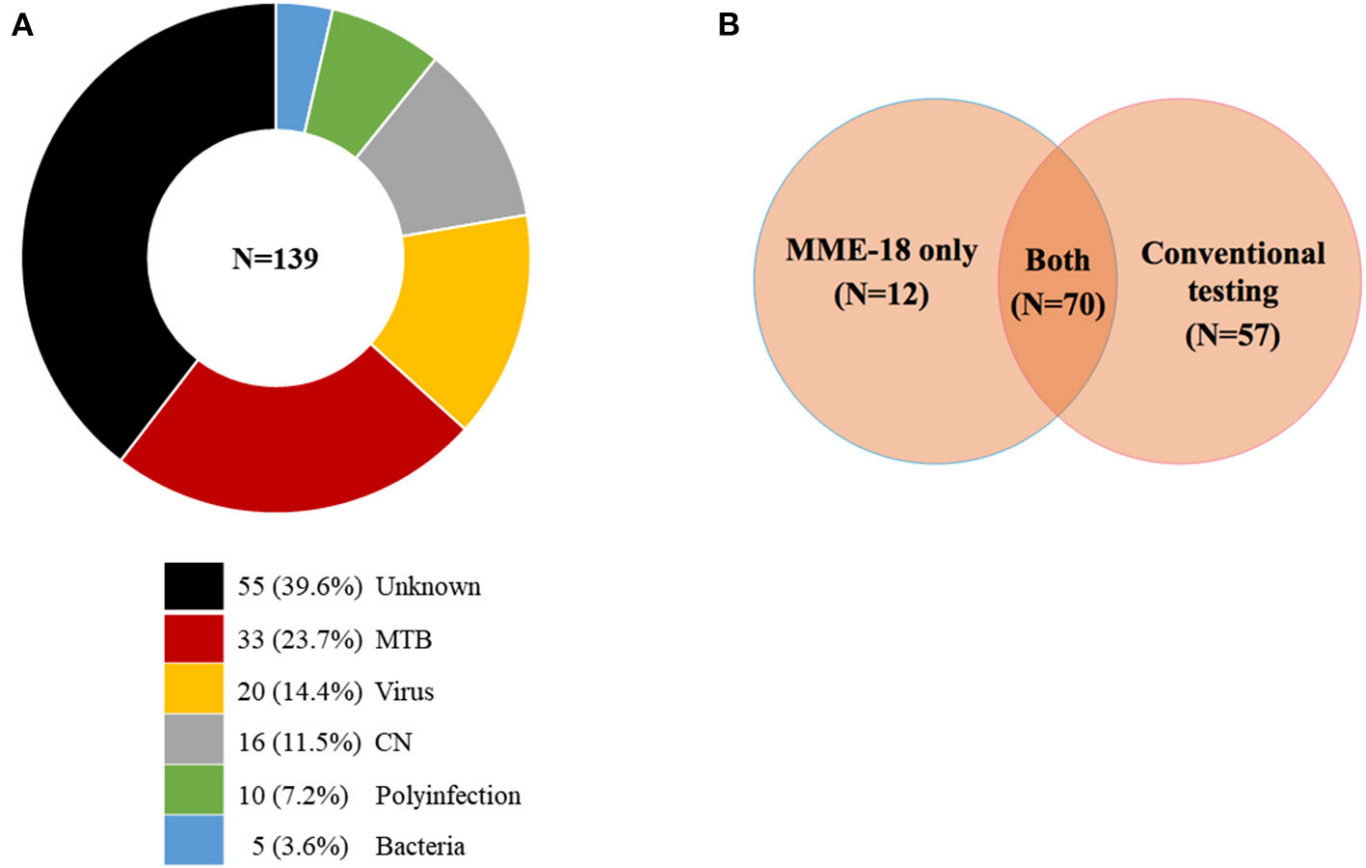
**(A,B)** The infections diagnosed by MME-18, conventional testing, and clinical evaluation.

As shown in Figure 2A and Table 2, all tested specimens were positive for at least 1 pathogen among 18 tested pathogens by the combination of MME-18, conventional testing and clinical evaluation. In mono infections, the most prevalent organism was MTB, found in 23.7% (33/139) of cases. Viruses, including EBV (11.5%, 16/139), EV (1.4%, 2/139), and CMV (1.4%, 2/139), were also frequently detected. CN was also found in 16 specimens (11.5%, 16/139). Other mono-assay targets, including LM, SP, AB, and HI, were detected in 2 or fewer specimens. As shown in Supplementary Table S1, poly-infections were observed in 10 specimens, representing 7.2% of all (10/139). MTB and other pathogens co-infections constituted 50.0% (5/10) of cases. Three cases were co-infected with MTB and CN. EBV and other pathogens co-infection accounted for 60.0% (6/10) of cases. These common organisms found in poly-infections were also the most prevalent pathogens detected in mono-infection. Among three patients infected with MTB and CN, one received anti-tuberculosis treatment followed by antifungal mediations, while the others only received antifungal medications (**Figure 4**).

**Table 2:** The detected pathogens from suspicious ME patients.

**Viral pathogen**	**All positive**	**Mono virus**	**Two viruses**	**Detection rate**	**Virus specific co-infection proportion**
MTB	38	33	5	26.6%	13.2%
EBV	22	16	6	15.8%	27.3%
CN	19	16	3	13.7%	15.8%
CMV	5	2	3	3.6%	60.0%
EV	2	2	-	1.4%	0.0%
SP	2	1	1	1.4%	50.0%
LM	2	2	-	1.4%	0.0%
HSV-1	2	-	2	1.4%	100.0%
HI	1	1	-	0.7%	0.0%
AB	1	1	-	0.7%	0.0%

ME, Infectious meningitis and encephalitis; MME-18, Multiplex PCR detection for 18 pathogens of ME; MTB, *Mycobacterium tuberculosis*; HI, *Haemophilus influenzae*; MP, *Mycoplasma pneumoniae*; SP, *Streptococcus pneumoniae*; AB, *Acinetobacter baumannii*; LM, *Listeria monocytogenes*; EV, Enterovirus; HSV-1, Herpes simplex virus-1; EBV, Epstein-Barr virus; CMV, Cytomegalovirus; CN, Cryptococcus neoformans.

### Comparison of MME-18 and conventional testing

Of 139 infected patients, 12 cases were diagnosed only by MME-18, 57 patients only by conventional testing, and 70 cases by both comparator tests and MME-18 (Figure 2B and Supplementary Table S2). Pathogens only detected by MME-18 included MTB (6/12, 50.0%), EV (1/12, 8.3%) CMV (1/12, 8.3%), AB (1/12, 8.3%), EBV (1/12, 8.3%), and CMV/EBV co-infection (2/12, 16.7%). These were patients with clinical suspicion, but conventional testing was negative or specific tests were not done. These were also patients with uninfectious symptoms and unknown etiology whose MME-18 was positive. Of 57 patients with negative MME-18 results, 55 of 57 were diagnosed based on clinical evaluation conformed with ME and antituberculous/antivirals were effective (Supplementary Table S3). False-negative cases of MME-18 were mainly diagnosed by serologic testing or RT-PCR for organisms in blood samples, for those the comparator tests for CSF were also negative. It included CMV, HSV-1, EV, and EBV infection. Furthermore, two patients were weakly positive for Xpert MTB/RIF, while the MME-18 results were negative. The consistent infections detected by both MME-18 and comparator testing included MTB (25/70, 35.7%), CN (16/70, 22.9%), EBV (15/70, 21.4%), LM (2/70, 35.7%), EV, SP, CMV, and HI were detected in only one specimen, respectively, 8 of 10 co-infection have been detected by MME-18 and another conventional testing.

### Comparison of MME-18 and Xpert MTB/RIF

**Figure 3:**
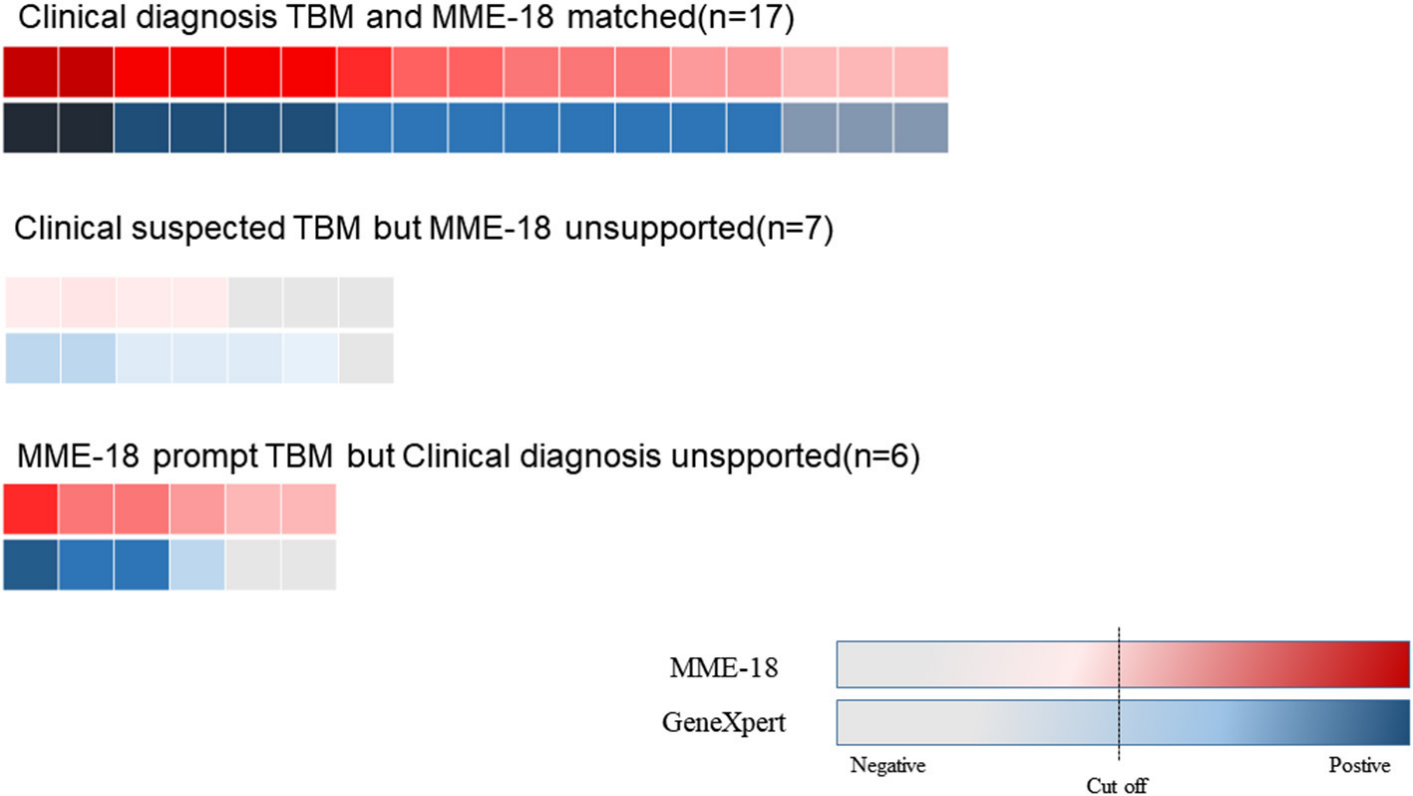
The comparison of MME-18 and Xpert MTB/RIF of suspected TBM. The consistent rate of MME-18 and Xpert MTB/RIF for the clinically diagnosed TBM was up to 100% (17/17). As for the five cases with solely positive MME-18 results, two cases were negative by Xpert MTB/RI. However, two patients were weakly positive for Xpert MTB/RIF among the seven clinically suspected TBM with negative MME-18 results.

After obtaining Xpert MTB/RIF results, we classified CSF samples in the following tuberculous meningitis (TBM) suspected categories: cases with both positive MME-18 results and conventional testing results in CSF; cases with positive MME-18 results but unsupported by clinical judgment; cases with clinical suspicion but unsupported with MME-18 results. The comparison results are shown in Figure 3 (The comparison of two methods for the detection of MTB showed in Supplementary Table S3). The consistency rate of MME-18 and Xpert MTB/RIF for the first category was up to 100%. Among cases with solely positive MME-18 results, two cases had negative Xpert MTB/RIF results. However, two patients were weakly positive for Xpert MTB/RIF, and the MME-18 results were negative.

### Clinical effect and feedback

**Figure 4:**
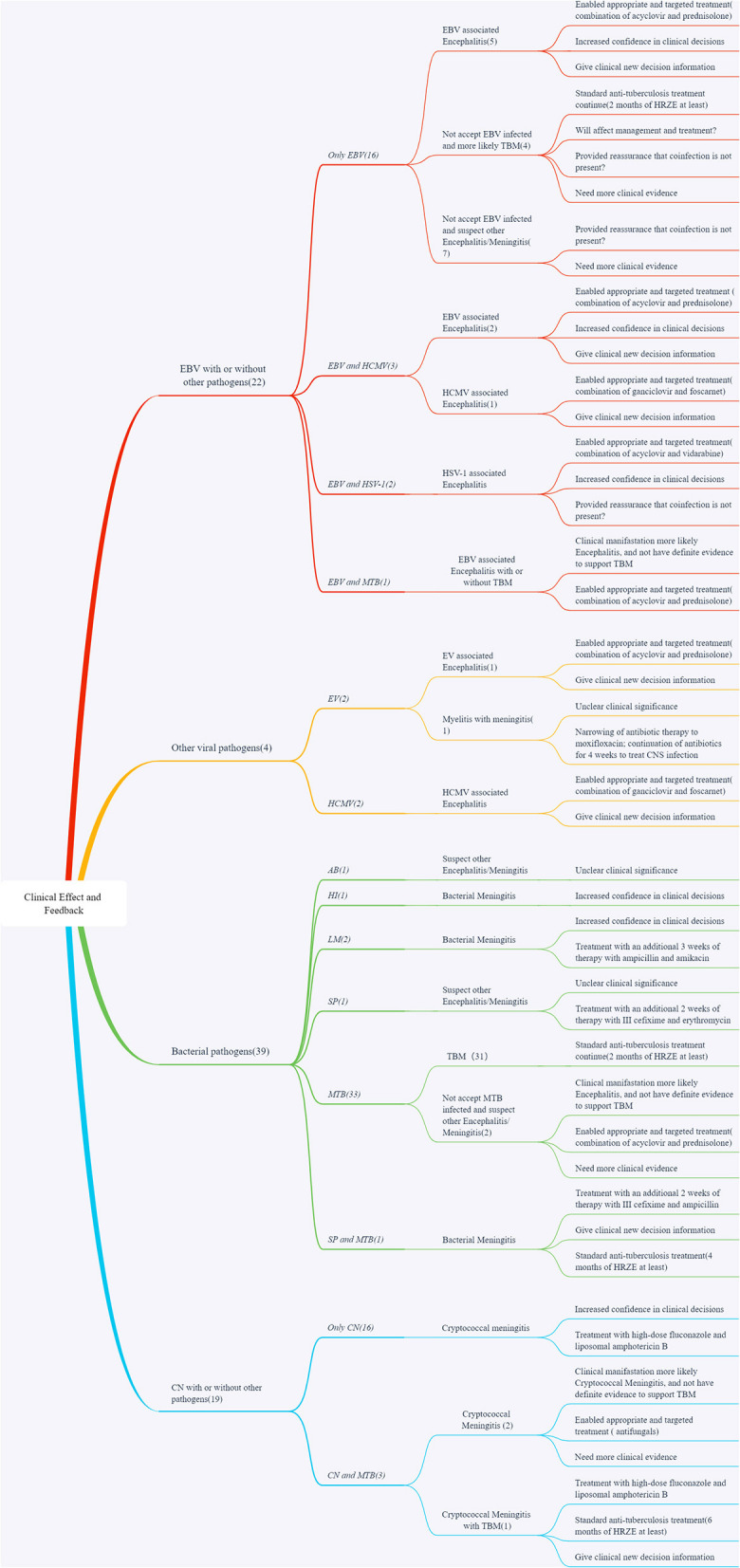
Clinical effect and feedback. ME, Infectious meningitis and encephalitis; MME-18, Multiplex PCR detection for 18 pathogens of ME; MTB, *Mycobacterium tuberculosis*; HI, *Haemophilus influenzae*; MP, *Mycoplasma pneumoniae*; SP, *Streptococcus pneumoniae*; AB, *Acinetobacter baumannii*; LM, *Listeria monocytogenes*; EV, Enterovirus; HSV-1, Herpes simplex virus-1; EBV, Epstein-Barr virus; CMV, Cytomegalovirus; CN, Cryptococcus neoformans.

The detailed clinical feedback and treatment are displayed in Figure 4. The concordance of MME-18 results with clinical diagnosis and treatment had four possible explanations. First, the MME-18 results confirmed clinical suspicions and thus enabled appropriate and targeted treatment (52/82, 63.4%). Second, MME-18 results increased confidence in clinical decisions which needed more clinical information and other techniques to confirm the judgment (13/82, 15.9%). Third, the MME-18 results could indicate new clinical findings, which might prompt clinicians with further proper confirmation tests and corresponding adjustments of healthcare management (14/82, 17.1%). Fourth, the clinical significance of MME-18 results might be unclear as the clinical characteristics induced by relevant pathogens detected by MME-18 were not apparent or some patients with co-infections were most sensitive to the treatment for the predominant pathogens (3/82, 3.7%). There were 96.3% (79/82) diagnoses made by MME-18 that had a favorable outcome, and two of twelve (EV and AB) diagnoses made solely by MME-18 had a likely unclear clinical significance.

## Discussion

As non-specific clinical symptoms disturb clinical judgment, clinicians rely on adjunctive tests. However, less sensitive and specific adjunctive tests also hamper the rapid and accurate diagnosis of ME. In this study, a multiplex PCR detection panel, MME-18, was utilized to investigate the epidemiological features of ME and the clinical effects of the results from the detection method. Our study identified 60.4% of patients who were positive for at least one causative agent in MME-18 or Xpert MTB/RIF. Among them, MME-18 was positive in 51.8% of cases, and in two cases with only positive Xpert MTB/RIF results. According to other studies in China, the identification of ME etiologies varies from 7.5 to 81.8% (14, 15, 27). In mono infections, the most are MTB (23.7%, 33/139), which also represents one of the most important etiological agents for ME. One nationwide surveillance study showed that the positive rate of MTB was 0.4‰ among 6,802 patients with acute ME (14), but another study reported the MTB positive rate was 7.7% among 350 patients in Shaanxi province (28). Twenty (14.4%) cases were positive for viruses (sixteen EBV, two CMV, and two EV), and there were 16 (11.5%) cases with positive CN. Furthermore, five patients were infected with bacteria (two LM, one HI, LM, and SP, respectively). In the same nationwide surveillance study, the positive detection rates were 18.4% (2,116/11,476) for EV, 1.55% (134/8,654) for SP, and 0.19% (16/8,417) for HI. Moreover, EV and HSV were the main cause among pediatric patients with ME and the main pathogens of bacterial ME is SP all over China. However, just two cases have HSV-I co-infected with EBV in this study. Some studies indicated that EV, HSV, SP, and MTB were common etiologies in ME in Asia (8–10), while in America and Europe, most ME patients are infected by HSV, SP, HI, and CN (11, 12, 29). The distribution of pathogens distribution among Chinese patients was different from other countries and regions. The regional epidemiologic differences of ME, limited sample size, diagnostic tests, and research duration may partly explain these contradictory findings, particularly in the epidemiological features of viruses. Further study with a larger sample is needed to explore in-depth the epidemiological features of ME, especially in Southwestern China.

For co-infection, EBV (60.0%, 6/10) co-infection was the most common etiology, and MTB (50.0%, 5/10) was the second in our study. Interestingly, EBV was also the most frequently detected virus in monoinfection. Additionally, we have identified 7.9% (3/38) MTB and CN co-infections of all MTB cases. This differed from the results of a large-scale publication of MTB and CN co-infection between 1993 and 2006 in Taiwan, which found 0.6% co-infection cases (23/4,053 total patients) (30). The discrepancy may be due to the low sensitivity of previous studies for co-infection. Some studies showed that the misdiagnosis rate was up to 76.6% due to non-specific clinical manifestations and low sensitivity of adjunctive tests (31). Therefore, MME-18 may have the advantage to detect poly-infection in CSF. We also reported MTB and CN co-infection in southwestern China, which has not been shown by a systematic review while the review concludes that the co-infections of MTB and CN were more popular in southern and eastern China (31). Poly-infections remind clinicians to adjust medications and treatment duration, differing from mono-infections (32–34). The detection of co-infections by MME-18 implies its value in detecting unexpected pathogens that clinicians may ignore due to indistinguishable manifestations or individual variations.

There has not been a ME panel utilizing multiplex PCR designed for Chinese people. Several studies used the FilmArray ME panel to detect ME (11, 12, 29, 35), which tested up to 14 pathogens but did not include MTB and JEV, which are prevalent among Chinese patients. Likewise, MME-18 can complete the test in <3.5 h at a low cost, as FilmArray (36). It shows that MME-18 can help diagnose infectious ME combined with routine microbiological tests. MME-18 is an effective tool for detecting common pathogens among Chinese patients. Furthermore, metagenomic next-generation sequencing (mNGS) is becoming increasingly available for pathogen detection directly from CSF. It enables the detection of various organisms with a single test without a priori selection of target pathogens (37). However, CSF mNGS testing alone cannot rule out infection. In addition, high costs (~$2,000–4,000), relatively long turnaround time ranges from 48 h to 2 weeks, low analytic sensitivity, and low negative predictive values hinder routine use of mNGS in the clinical laboratory (38). The novel MME-18 proved timely and accurate in this study. Furthermore, given the high prevalence of MTB in China, especially in the Southwestern part of the mainland, here we performed Xpert MTB/RIF in the CSF samples of suspected TBM cases. Xpert MTB/RIF has been formally recommended by the WHO for the diagnosis of tuberculosis, with a sensitivity of approximately 60% [43]. In our study, the total consistency between MME-18 and Xpert was 89.5%, revealing the potential value of MME-18 in the diagnosis of TBM. Considering the inconsistency of the two testing results, clinical evaluation is needed before making therapeutic decisions. Still, these two test methods are helpful for the rapid screening and detection of TBM. For other common pathogens, MME-18 detected more pathogens than comparator methods in our study. Although 56 cases were only detected by conventional testing, including serologic testing, PCR, CSF culture, blood culture, and sputum culture. The false negative results of MME-18 in these cases may be explained by the lower abundance of pathogens in CSF or the higher abundance of pathogens in other non-CSF specimens. The other case was detected by NGS, with a positive result for *Klebsiella oxytoca*.

It is noteworthy that detected pathogens in laboratory wether contribute to clinical diagnosis and treatment. After investigation of clinical feedback of the 12 cases without support of conventional testing, we investigated the potential cause for the inconsistency. First, we ruled out the possibility of sample-to-sample contamination, which frequently leads to false-positive results. Second, all the positive results of MME-18 have been verified by sequencing or RT-PCR. These results mean that the MME-18 has a possible high sensitivity to certain pathogens and further investigation is needed. The cases with only positive MME-18 may were suspected of other encephalitis/meningitis and need more clinical evidence to support the result of MME-18, while specific testing was lacking. The other potential cause is that clinicians considered the significant and clinical role of the pathogens tested by MME-18 from patients' CSF to be unclear. In our study, three cases with positive pathogens, while clinicians did not treat them with targeted treatment. One patient was positive for EV, and clinicians decided to administer moxifloxacin for 4 weeks. In addition, there was 1 patient positive for EBV and CMV, and clinicians ultimately prescribed cefotaxime for 3 weeks. One patient was positive for SP, which may have been from contamination during the sample collection process. Intriguingly, of the inconsistency of the result between clinicians and MME-18 for EBV, the most popular virus in this study, some researchers found that EBV can be persistent in some EBV-seropositive individuals without meningitis or encephalitis (39). Consistent with our results, EBV DNA is positive in concurrent infection with other viral or bacterial infections organisms (40).

However, our study has some limitations. The limited scale of our investigation may not elaborate on the characteristic of the distribution of causative agents in CSF. Therefore, our study was unable to represent the comprehensive prevalence and features of ME in southwestern China. Still, the performance of MME-18 still needs to be validated systematically in a larger cohort.

In conclusion, ME needs more attention and the distribution of pathogens among Chinese people differs from other countries and regions. MME-18 is an effective testing method for detecting pathogens in CNS infections. MME-18 should be optimized and validated by future studies. The combination of MME-18, conventional testing methods, and clinical evaluation can maximize the detection rate and diagnostic yield in ME.

## Data availability statement

The raw data supporting the conclusions of this article will be made available by the authors, without undue reservation.

## Ethics statement

The studies involving human participants were reviewed and approved by the Institutional Review Board of the West China Hospital. The patients/participants provided their written informed consent to participate in this study.

## Author contributions

YS and WH wrote the manuscript and participated in the experiment all the way. SG provided professional evaluation and consultant. XW participated in the analysis of data. MT engaged in the acquisition of data (laboratory or clinical). MW and BY designed and supervised the study. All authors contributed to the article and approved the submitted version.
