# Supplementary material for: Multiplex detection of meningitis and encephalitis pathogens: A study from laboratory to clinic
Source: Front Neurol. 2022 Dec 16;13:1054071. doi: 10.3389/fneur.2022.1054071 (PMC9800896; doi:10.3389/fneur.2022.1054071)
Supplement: Supplementary file 1 [file Data_Sheet_1.docx]

**Supplementary materials**

Figure S1. The demonstration of Nucleic acid extraction and MME-18 testing

Table S1. Polyinfection types of double and triple pathogens

| Number | Type | Cases |
| --- | --- | --- |
| 1 | MTB+CN | 3 |
| 2 | MTB+SP | 1 |
| 3 | MTB+EBV | 1 |
| 4 | EBV+CMV | 3 |
| 5 | EBV+HSV-1 | 2 |
| Total | - | 10 |

MTB, Mycobacterium tuberculosis; CN, Cryptococcus neoformans; SP, Streptococcus pneumoniae; HSV-1, Herpes simplex virus-1; EBV, Epstein-Barr virus; CMV, Cytomegalovirus.

Table S2. Diagnosis of 57 patients with meningitis and encephalitis with negative MME-18 results.

| Target | MTB  (n/nt)^a^ | CMV  (n/nt) | EBV  (n/nt) | EV  (n/nt) | HSV-1  (n/nt) | Unknown |
| --- | --- | --- | --- | --- | --- | --- |
| MME-18 | 0/57 | 0/57 | 0/57 | 0/57 | 0/57 | - |
| CSF cultures | 0/35 | - | - | - | - | - |
| PCR for pathogens in CSF | 0/50 | - | - | - | - | - |
| PCR for pathogens in plasma | - | 3/10 | 2/12 | - | - | - |
| Xpert MTB/RIF | 2/30 | - | - | - | - | - |
| Acid fast stain | 0/35 | - | - | - | - | - |
| **Serology tests** |  |  |  |  |  |  |
| CMV IgM | - | 2/20 | - | - | - | - |
| CMV IgG | - | 18/20 | - | - | - | - |
| EBV IgM | - | - | 0/15 |  |  | - |
| EBV IgG | - | - | 14/15 |  |  | - |
| HSV-1/2 IgM | - | - |  | - | 7/10 | - |
| HSV-1/2 IgG | - | - |  | - | 4/10 | - |
| Clinical evaluation | 9/57 | - | - | - | - | 46/57 |

^a^ Number of positive tests/number of total tested; MME-18, Multiplex PCR detection for 18 pathogens of meningitis and encephalitis.; HSV-1, Herpes simplex virus-1; EBV, Epstein-Barr virus; CMV, Cytomegalovirus.

Table S3. Comparison of Xpert MTB/RIF and MME-18 for the diagnosis of tuberculous meningitis

| Assay | MME-18 | Xpert MTB/RIF |
| --- | --- | --- |
| Description | PCR fragment analysis combined with capillary electrophoresis. | Cartridge-based PCR |
| CSF | 200~1000ul | 1000ul~ |
| Time to results | 3.5h | 2.5h |
| Limit of Detection | 4 copies/uL or 20 copies/rxn | requiring five genome copies of purified DNA per reaction or 131 cfu/ml |
| Advantages | Fast, nearly as sensitive as culture, specific, 18 common pathogens multiple detection | Quick, similar sensitivity to culture, specific, ease of use, drug-resistant Tuberculosis |
| Disadvantages | Cost, lab expertise, lab apparatus | Cost, requires significant  infrastructure, limited shelf life on  cartridges |

Assay is meant to describe a test category, not a specific commercial test; CSF, Cerebrospinal Fluid. MME-18, Multiplex PCR detection for 18 pathogens of meningitis and encephalitis.
