# Supplementary figures and images for: Multiplex detection of meningitis and encephalitis pathogens: A study from laboratory to clinic
Source: Front Neurol. 2022 Dec 16;13:1054071. doi: 10.3389/fneur.2022.1054071 (PMC9800896; doi:10.3389/fneur.2022.1054071)

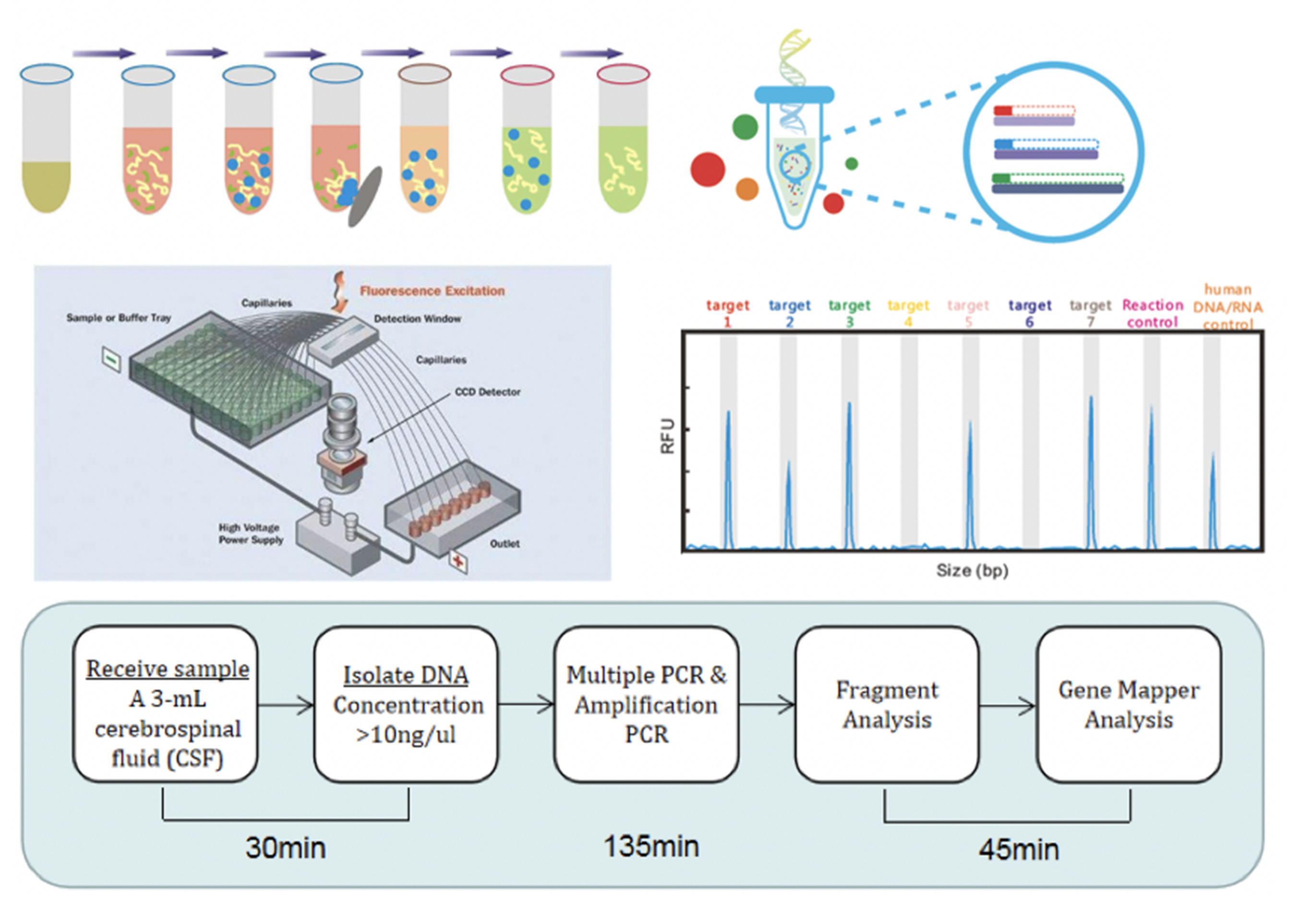

Supplement: Supplementary file 2 [file Image_1.TIFF]
